# 

**Published:** 1878-11

**Authors:** 


### OHIO STATE DENTAL SOCIETY.

The annual meeting of the Ohio State Dental Society will be held in Columbus, beginning on Tuesday, December 3d, at two o'clock p. m. We anticipate a large attendance and a good time for thorough and efficient work.

It is desirable that the profession of the State be fully represented. There ought to be an attendance of at least four hundred at this meeting.

Let *all* come who possibly can, and every one be prepared to add something of interest and profit to those present. The following are the subjects for consideration and discussion at the meeting, viz:

1. Histology of the teeth.

2. Nodular calcification of the tooth pulp; its diagnosis and treatment.

3. Physical diagnosis of diseased action arising from, or in connection with, the dental organs,

4. Dental mechanism.

5. Heredity; as its influence is manifested on or by the teeth.

6. The health and vital force of the dentist.

*a.* What can be done to avoid a disturbance of mental equanimity and nervous irritation of the operator.

*b.* What hygenic and therapeutic means will best conserve the general health of the dentist.

7. Galvanism of the mouth.

8. Abrasion of the teeth; sensitiveness of necks; causes and treatment.

Now it is desirable that every member and every one who intends to become a member, prepare himself for taking part in the consideration of each of these subjects. Each one should commit his thoughts to paper beforehand, that he might have them for use at the time of need.

Let every new or improved instrument or appliance be brought for inspection.

The dental profession of Ohio occupies an enviable position, and that can not be maintained without effort, and it is all-important that this effort be not wanting. Let there be such an outpouring as has not been seen hitherto. Come one, come all.

### IMPROVEMENTS.

*THE DENTAL REGISTER,*

A MONTHLY MAGAZINE DEVOTED TO THE INTERESTS OF

THE DENTAL PROFESSION.

*Terms Reduced to $2 50 per Year. Single Copies 25c.*

*EDITED BY PROF. J. TAFT, D. D. S.,*

AND PUBLISHED BY

SPENCER & CROCKER, Ohio Dental Depot.

The volume commences in January and closes in December.

The Register being issued monthly is always fresh, therefore Indispensable to the practitioner who desires to keep posted up with the new inventions and improvements which are daily developed. Neither expense nor effort will be spared to give value and variety to its contents. Articles will be illustrated with well executed engravings whenever they will add to the interest or assist to explanation.

Its extensive circulation and frequency of issue make it one of the BEST DENTAL ADVERTISING MEDIUMS in the United States.

All subscrptions must begin with January or July.

Local or special notices, five lines of less, will be inserted for $3.00 each insertion; over five lines, 50 cts. per line.

All advertising bills payable in advance, or at furthest, after the issue of the first number containing the advertisement. All transient advertisements to be paid for in advance.

☞CONTRIBUTIONS SOLICITED.

Exclusively Editorial Communications should be addressed to Editor.

The latest number of the Register may be ordered with or without other goods at any time, and all letters should be addressed to

**SPENCER & CROCKER, Ohio Dental Depot, Cincinnati, O.**

